# Registry study on failure incidence in 1,127 revised hip implants with stem trunnion re-use after 10 years of follow-up: limited influence of an adapter sleeve

**DOI:** 10.1080/17453674.2019.1618649

**Published:** 2019-06-18

**Authors:** Saverio Affatato, Monica Cosentino, Francesco Castagnini, Barbara Bordini

**Affiliations:** a Laboratorio di Tecnologia Medica, IRCCS—Istituto Ortopedico Rizzoli, Bologna, Italy;; b Ortopedia-Traumatologia e Chirurgia protesica e dei reimpianti d’anca e di ginocchio, IRCCS—Istituto Ortopedico Rizzoli, Bologna, Italy

## Abstract

Background and purpose — Little is known about the role of retained trunnions in revision hip arthroplasties, i.e., when only the femoral head is substituted. Wear (fretting corrosion) and ceramic head fractures are 2 poorly understood concerns related to use, and the role of adapter sleeves has not been defined. In this registry study we assessed the influence of sleeve interposition on re-revision rates in revision hip arthroplasties with retained stems. Confounding factors (demographics, implant-related features) and failures were also analyzed.

Patients and methods — We conducted a registry study on 1,127 revised implants (retained trunnion and head exchange). In 26% of implants an adapter sleeve was interposed; in 74% no adapter sleeve was implanted. Demographic and implant-related features were investigated including a descriptive analysis of failures.

Results — The mean follow-up of revised implants with and without the use of an adapter sleeve was 3.3 and 5.1 years, respectively. The implant survival without an adapter sleeve was significantly higher, 98.4% (95% CI 96.9–99.8) vs. 95.2% (CI 93.2–96.6) with an adapter sleeve at 5 years. No re-revisions due to adverse local tissue reactions or ceramic head fractures were reported. In order to overcome the different distribution of head materials and head sizes in the two cohorts, only Delta balls were investigated.

Interpretation — Adapter sleeve interposition had a minor influence on the revision rates. No adverse local tissue reactions or head fractures occurred.

Retained modular head–neck junctions in revision hip arthroplasty imply 2 main concerns: wear and ceramic head failure (Hannouche et al. 2010, Gührs et al. 2015, Higgs et al. 2016, Osman et al. 2016). Fretting and mechanically assisted crevice corrosion may occur at the head–neck junction in case of metal-on-metal contact, potentially leading to metallic ion release, adverse local tissue reactions, and implant failures may ensue (Osman et al. 2016, Koch et al. 2017, MacDonald et al. 2017). In particular, mixed metal couplings have been reported to increase corrosion and fretting (Koch et al. 2017).

Concerning ceramic balls, trunnion wear may act as a stress enhancer, initiating cracks that, eventually, would lead to head fracture (Hannouche et al. 2003, 2010). Therefore, when the stem is retained and ceramic heads are implanted, producers advise surgeons to interpose a titanium adapter sleeve to improve contacts between the conical trunnion of the femoral stem and the conical bore of the femoral head (Koch et al. 2017). However, with the exception of a few in vitro studies concerning adapter sleeve use (Gührs et al. 2015, Koch et al. 2017, MacDonald et al. 2017, Berstock et al. 2018), little is known about the influence on implant survivorship of retained trunnions at revision, with or without the interposition of adapter sleeves. Clinical impacts of wear and ceramic head fractures due to trunnion re-use appeared negligible, regardless of the interposition of an adapter sleeve, with only anecdotal reports of head fractures and metallosis (Pulliam and Trousdale 1997, Hannouche et al. 2010, Koch et al. 2017).

We assessed with registry data the influence of adapter sleeves on re-revision rates, and the role of demographics and implant-related features on survivorship.

## Patients and methods

The Register of Prosthetic Orthopedic Implants (RIPO) is a regional database (surveillance over 4,500,000 inhabitants) recording clinical conditions of patients, surgical procedures, implant characteristics, i.e., fixation, head size and material, and type of acetabular cup and stem (RIPO 2018, Bordini et al. 2019). Primary and revision hip and knee replacement surgeries are included. All hospital admissions of Emilia Romagna residents, even when occurring in other regions, are paid for by the region itself, and thus recorded in the registry. Conversely, patients undergoing arthroplasty procedures in Emilia Romagna but living outside the region were excluded from the analysis, as these patients had no stable connections with the regional health system (Bordini et al. 2019). In the RIPO we identified all cases with stem retention and head exchange during 2000–2016.

### Statistics

Patients’ ages were compared using a t-test. Sex and implant-related features were compared using chi-square analysis. The survival rate of patients was calculated and plotted according to the Kaplan–Meier method. The end-point was stem or neck revision, whereas revision of the cup/insert was not considered as a failure, since it did not involve stem issues. Implants were followed until the last date of observation (date of death or December 31, 2016). Statistical analyses were performed using SPSS 14.0, version 14.0.1 (SPSS Inc, Chicago, IL, USA) and JMP, version 12.0.1 (SAS Institute Inc, Cary, NC, USA, 1989–2007).

### Ethics, funding, and potential conflicts of interests

Ethical approval was not necessary due to the features of registries and databases (data anonymization). No funds were received for this study. All authors declare that no potential conflict of interest exists.

## Results

1,127 cases with revision implants were included in the study. 2 cohorts were identified, with and without interposition of an adapter sleeve. The demographic features of the 2 cohorts were similar (Table 1). The vast majority of stems were made of titanium alloy and had a 12/14 taper. The main differences in the 2 cohorts were related to head size (larger in the “adapter sleeve” cohort) and head material (more ceramic heads in the “adapter sleeve” cohort, more CrCo heads in the “no adapter sleeve” cohort) (p = 0.001).

**Table 1.** Demographics and implant-related features of the 2 cohorts (with and without adapters). Values are frequency (%) unless otherwise specified

Descriptive	Adapter	No adapter	
Number of revised implants	296 (26.3)	831 (73.7)	
Mean age at revision (range)	69 (25–89)	71 (29–92)	
Sex			
	Female	177 (60)	533 (64)
	Male	119 (40)	298 (36)
BMI group **^a^**			
	Underweight	3 (1)	9 (1)
	Normal	87 (36)	262 (39)
	Overweight	37 (15)	129 (19)
	Obese	118 (48)	279 (41)
Stems **^b^**			
	CONUS Sulzer (Ti6Al7Nb)	26 (9)	64 (8)
	CBC Mathys (Ti6Al4V)	25 (8)	39 (5)
	ANCA FIT Cremascoli (Ti6Al4V)	35 (12)	21 (2)
	CLS Sulzer (Ti6Al7Nb)	15 (5)	39 (5)
	TAPERLOC Biomet (Ti6Al4V)	15 (5)	29 (3)
	SL PLUS Endoplus (Ti6Al7Nb)	5 (2)	38 (5)
	VERSYS FIBER METAL TAPER		
	Zimmer (Ti6Al4V)	6 (2)	25 (3)
	CONUS Zimmer (Ti6Al7Nb)	5 (2)	25 (3)
	CORAIL Depuy (Ti6Al4V)	5 (2)	25 (3)
	ABGII Howmedica (TMZF)	3 (1)	25 (3)
	CFP Link (Ti)	6 (2)	16 (2)
	CLS Zimmer (Ti6Al7Nb)	6 (2)	15 (2)
	SPS Symbios (Ti6Al4V)	9 (3)	11 (1)
	Others (models with < 20 cases)	131 (45)	454 (55)
Fixation			
	Cementless	271 (92)	677 (82)
	Cemented	22 (8)	150 (18)
Neck^c^			
	Fixed	218 (74)	731 (88)
	Modular	78 (26)	100 (12)
Head size			
	< 28 mm	–	10 (1)
	28 mm	57 (19)	442 (56)
	32 mm	68 (23)	164 (21)
	36 mm	143 (48)	157 (19)
	≥ 38 mm	28 (10)	21 (3)
Head material^d^			
	Biolox Delta	268 (91)	160 (20)
	Biolox Forte	3 (1)	85 (11)
	Ceramys	–	5 (1)
	Inox Stainless Steel	16 (5)	66 (8)
	CrCo(Mo)	9 (3)	444 (56)
	Oxinium	–	37 (4)
Number of failures (stem + neck)	5 (1.7)	39 (4.7)	
Number of failures (stem)	3 (1.0)	36 (4.3)	

a Missing data: 203 (18% of the total).

b Alloy of the cementless implant, all tapers are 12/14 except ABGII (V40) — missing data: 9 (1% of the total).

c Missing data: 37 (3% of the total).

d Missing data: 34 (3% of the total).

**Figure 1.**
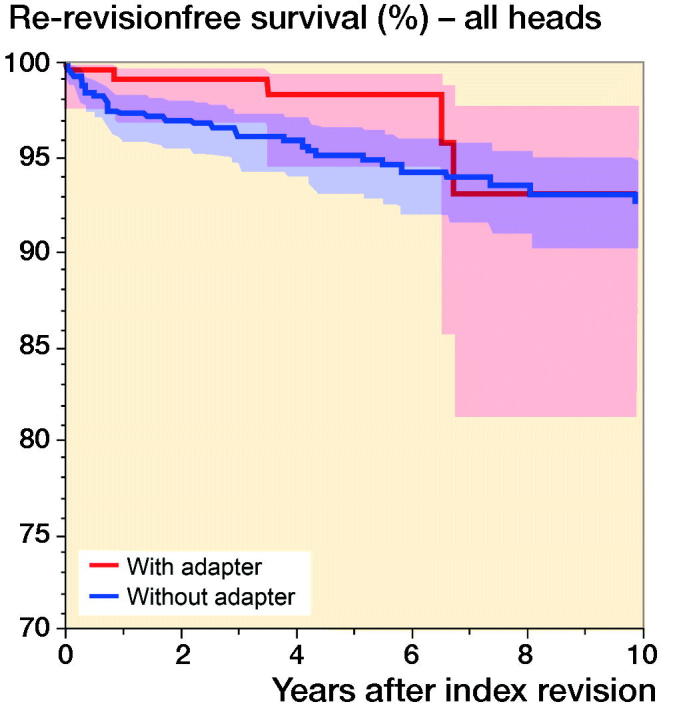
Kaplan–Meier survival rates of the 2 cohorts (with and without adapters).

The implant survival curves of the 2 cohorts showed a statistically significant difference (p = 0.04), with revision implants with adapter sleeve interposition performing better (Figure 1).

The most frequent reason for re-revision was stem aseptic loosening in both cohorts (Table 2).

**Table 2.** Reasons for re-revision. Values are frequency (%)

Reason	Adapter	No adapter
Stem aseptic loosening	2 (0.7)	9 (1.1)
Modular neck breakage	2 (0.7)	–
Septic loosening	–	8 (1.0)
Total aseptic loosening	–	6 (0.7)
Periprosthetic bone fracture	–	5 (0.6)
Recurrent prosthesis dislocation	–	4 (0.5)
Cup aseptic loosening	–	4 (0.5)
Pain without loosening	–	1 (0.1)
Unknown	1 (0.3)	2 (0.2)
Total	5 (1.7)	39 (4.7)

Re-revisions due to sepsis occurred only in the “no adapter sleeve” cohort (1.0%). No ceramic head fracture occurred. No demographic or implant-related factor apparently influenced re-revision rates or reasons for re-revision (p = 0.4). Of the 44 cases, 18 were in overweight patients, and 6 were obese. 22 failures were CrCo head implants (5% of the total CrCo head implants): all these cases were without an adapter sleeve and a 28 mm head was frequently implanted (in 16/22 cases). 8 failures involved BIOLOX-Delta heads (1.9%) (CeramTec GmbH, Plochingen, Germany). 9/88 BIOLOX-Forte heads (CeramTec GmbH, Plochingen, Germany) failed: all failures were implanted without an adapter sleeve; 5 failures involved 28 mm heads. Most of the failures occurred in cementless, titanium stems with 12/14 tapers (the most represented cases in both cohorts).

**Figure 2.**
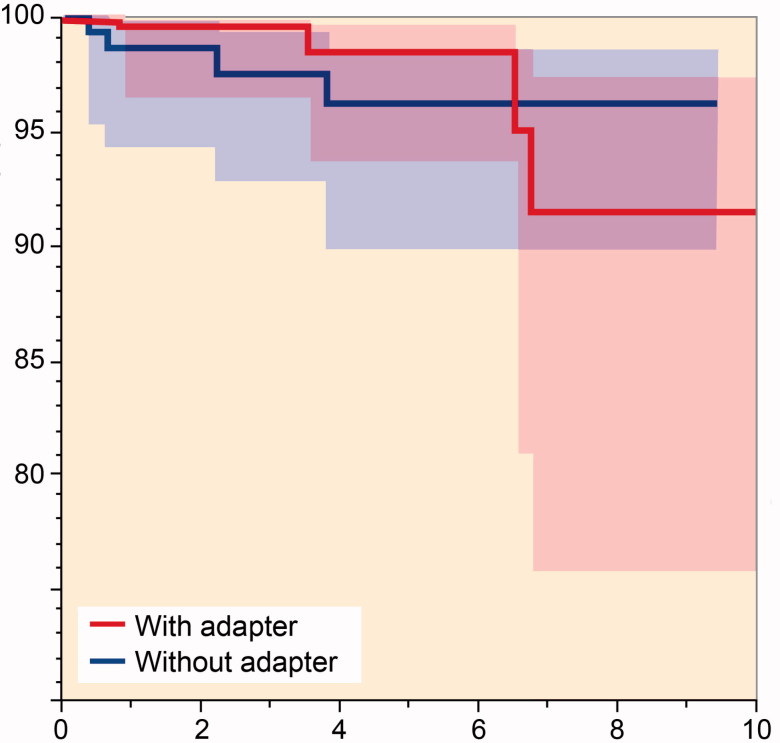
Kaplan–Meier survival rates of the 2 cohorts (with and without adapters) involving only the Delta head.

However, this comparison is affected by a different distribution of 2 notable implant-related variables, head size and head material, which were strongly connected (as CrCo heads were frequently 28 mm). Thus, in order to control these confounding factors a further analysis was performed, involving only Delta heads. The “adapter sleeve” cohort (268 implants at a mean follow-up of 3.2 years [0–11]) achieved a lower survivorship than the “no adapter sleeve” cohort (60 implants at a mean follow-up of 3.6 years [0–9.4]), (Figure 2); a non-statistically significant difference was observed (p = 0.2). 4 failures occurred in both groups. Middle-aged, overweight/obese men were more commonly involved, regardless of the use of adapter sleeves (Table 3, see Supplementary data).

## Discussion

The purpose of this study was to investigate the role of adapters in revision hip arthroplasties with retained stems. We found that the use of adapter sleeves statistically significantly reduced the rate of re-revisions. However, no cases of re-revisions due to metallosis or head fractures were reported. Moreover, the 2 groups differed strongly in terms of head materials and head size. When only Delta heads were involved, the re-revision rates were similar between the “adapter sleeve” and “no adapter sleeve” cohorts. Thus, the role of adapter sleeves seems clinically negligible at mid-term follow-up. Indeed, the current pertinent literature involving clinical evaluations tends to support this finding. Hannouche et al. (2010) performed a retrospective investigation on the fracture risk of ceramic heads implanted on retained trunnions without the use of an adapter sleeve. Their investigation was limited to 61 revised hip implants, following the follow-up of alumina–alumina primary THAs. The authors observed no fractures and suggested replacing the ceramic head when the femoral stem was fully integrated. Kim et al. (2018) performed a prospective study to assess the prevalence of ceramic head fractures on retained trunnions without interposition of titanium adapter sleeves in 100 implants of the same material and design. They found no cases of ceramic head fractures, ascribed to the relatively pristine trunnion surface—assessed before fixing the new head. Moreover, the authors suggested that a titanium adapter sleeve was not necessary with minimal fretting and/or corrosion scores.

There were 2 notable findings in our study. The first was that without sleeve interposition high rates of failure occurred with 28 mm CrCo heads and with Forte heads. It is likely that mechanical (high rates of 28 mm head involvement) and tribological features (higher wear in CrCo heads) were responsible, rather than adapters, as no re-revisions were due to suspicious adapter sleeve failure. The second was that septic failures occurred only in the “no adapter sleeve” cohort, where there was a higher rate of CrCo heads with no adapter sleeve interposition, implanted on titanium stems. Metal-on-metal interfaces are more prone to infections, probably due to necrosis and immunomodulation induced by metal particle release (Bordini et al. 2019). Mixing metals may even worsen the local situation. Thus, taper wear as a generator of local metal ion could occur in these cases. However, these speculations require in vitro testing and large observational studies.

Strengths of our study are mainly related to the large numbers of implants involved. Our study has a number of limitations. First, the study involved a large number of different materials, designs, and taper sleeve implant techniques based on surgeon personal criteria. Second, only limited follow-ups were achieved and it was not possible to take account of the level of activity of the patients. Third, visual inspection of tapers and surrounding soft tissues was lacking as offset evaluation, due to the large number of stem designs. In addition, CrCo cohorts with and without adapter sleeves could not be compared, as there were only 9 CrCo head implants with adapter sleeve interposition.

In summary, the interposition of an adapter sleeve seemed only to influence re-revision rates slightly. The 2 main concerns related to trunnion re-use, wear and ceramic fractures, did not occur in this registry study. Possible adverse local tissue reactions due to metal release could not be completely ruled out by the registry studies, as no visual inspection and histology were available. We found a high rate of 28 mm CrCo head implant failures when no adapter sleeves were used. Similarly, a very high rate of Forte head failures on retained, “uncovered” tapers occurred. These 2 conclusions are only merely speculative (no clear association with lack of adapter sleeve), but may be a matter for further investigation.

## Supplementary data

Table 3 is available as supplementary data in the online version of this article, http://dx.doi.org/10.1080/17453674.2019. 1618649

SA and BB performed the conception and design of the study. SA and FC wrote the main manuscript text. MC and BB performed the statistical analysis and interpretation of data. MC and BB prepared Figure 1. All authors reviewed the final version of the manuscript.

*Acta* thanks Bart Bosker and Timothy Wrightfor help with peer review of this study.

**Table:** 

	Years after index revision				
At risk	0	1	3	5	7
Adapter	296	227	137	79	30
No adapter	831	688	515	371	248

**Table:** 

	Years after index revision				
At risk	0	1	3	5	7
Adapter	268	204	121	66	22
No adapter	160	127	89	45	20

## Supplementary Material

Supplemental Material
