# Supplementary material for: Registry study on failure incidence in 1,127 revised hip implants with stem trunnion re-use after 10 years of follow-up: limited influence of an adapter sleeve
Source: Acta Orthop. 2019 Jun 18;90(5):417–20. doi: 10.1080/17453674.2019.1618649 (PMC6746291; doi:10.1080/17453674.2019.1618649)
Supplement: Supplemental Material [file IORT_A_1618649_SM3466.pdf]

Supplementary data

Table 3. Description of the 44 failures

Stem fixation / Head size and material	Sex	BMI	Reason for revision [a]	Age at revision	Reason for re-revision [a]	Age at re-revision	Stem type	Stem years in situ
**No adapter**								
Cemented								
28 mm								
CrCo	F	Overweight	Cup loosening	79	Dislocations	79	SPECTRON Smith & Nephew (CrCo, 12/14)	6.0
	M	Overweight	Cup loosening	80	Global loosening	81	SPECTRON Smith & Nephew (CrCo, 12/14)	12.3
SS	M	Normal	Dislocations	76	Periprosthetic fract.	76	LC Samo (CrCoMo, 12/14)	0.6
32 mm								
CrCo	M	Normal	Periprosthetic fract.	76	Cup loosening	76	LC Samo (CrCoMo, 12/14)	6.0
Oxinium	F	Overweight	Liner failure	80	Stem loosening	81	BASIS Smith & Nephew (CrCo, 12/14)	11.6
36 mm								
CrCo	F	Unavailable	Unavailable	73	Cup loosening	81	ALBI PTC Cremascoli (Ti6Al4V, 12/14)	14.3
Cementless								
28 mm								
Ceramys	F	Unavailable	Dislocations	79	Dislocations	79	CBC Mathys (Ti6Al4V, 12/14)	0.8
CrCo	F	Obese	Dislocations	68	Global loosening	71	SL PLUS Endoplus (Ti6Al7Nb, 12/14)	7.7
	F	Obese	Primary instability	71	Septic loosening	71	RECTA Adler Ortho (Ti6Al4V, 12/14)	0.7
	F	Overweight	Cup loosening	74	Septic loosening	74	SL PLUS MIA (Ti6Al7Nb, 12/14)	0.0
	F	Overweight	Dislocations	41	Stem loosening	42	CORAIL DEPUY (Ti6Al4V, 12/14)	1.8
	F	Overweight	Pain	73	Periprosthetic fract.	77	HYDRA Adler Ortho (Ti6Al4V, 12/14)	5.5
	F	Overweight	Periprosthetic fract.	64	Unavailable	65	SL PLUS ENDOPLUS (Ti6Al7Nb, 12/14)	0.9
	F	Unavailable	Dislocations	81	Periprosthetic fract.	81	CLS Sulzer (Ti6Al7Nb, 12/14)	9.8
	F	Unavailable	Head failure	52	Global loosening	62	STEM CREMASCOLI (Ti6Al4V, 12/14)	11.7
	M	Normal	Septic loosening	74	Stem loosening	80	PPF Stratec (Ti6Al4V, 12/14)	6.6
	M	Obese	Cup loosening	65	Periprosthetic fract.	66	ABGII Howmedica (TMZF, V40)	3.5
	M	Obese	Pain	70	Stem loosening	71	EHS Cremascoli (Ti6Al4V, 12/14)	6.1
	M	Unavailable	Cup loosening	71	Septic loosening	75	CONUS Sulzer (Ti6Al7Nb, 12/14)	7.6
	M	Unavailable	Dislocations	68	Unavailable	76	CLS Sulzer (Ti6Al7Nb, 12/14)	10.2
	M	Unavailable	Unavailable	50	Septic loosening	53	CONUS Sulzer (Ti6Al7Nb, 12/14)	10.6
Forte	F	Normal	Cup loosening	64	Periprosthetic fract.	64	AD Samo (CrCoMo, 12/14)	3.4
	F	Normal	Dislocations	82	Dislocations	82	CLS Sulzer (Ti6Al7Nb, 12/14)	9.2
	F	Normal	Head failure	72	Periprosthetic fract.	78	SPS Symbios (Ti6Al4V, 12/14)	10.6
	F	Obese	Pain	52	Septic loosening	53	CONUS Zimmer (Ti6Al7Nb, 12/14)	1.7
	F	Unavailable	Unavailable	65	Cup loosening	68	ANCA FIT Cremascoli (Ti6Al4V, 12/14)	4.0
Oxinium	M	Overweight	Pain	70	Dislocations	71	SL PLUS Endoplus (Ti6Al7Nb, 12/14)	3.0
32 mm								
CrCo	F	Unavailable	Dislocations	57	Global loosening	60	PERFECTA MicroPort (Ti6Al4V, 12/14)	3.5
	M	Overweight	Head failure	63	Global loosening	70	PROFEMUR Z Cremascoli (Ti6Al4V, 12/14)	8.8
Forte	F	Overweight	Unavailable	74	Septic loosening	74	MINIMAX Medacta (Ti6Al7Nb, 12/14)	1.2
36 mm								
CrCo	F	Unavailable	Cup loosening	68	Global loosening	73	CBC Mathys (Ti6Al4V, 12/14)	9.2
	M	Unavailable	Unavailable	79	Septic loosening	80	SL PLUS MIA (Ti6Al7Nb, 12/14)	0.4
Delta	M	Obese	Cup loosening	60	Septic loosening	60	CORAE Adler Ortho (Ti6Al4V, 12/14)	1.2
	M	Overweight	Cup loosening	61	Pain	65	CONELOCK SHORT Biomet (Ti6Al4V, 12/14)	7.0
	M	Overweight	Pain	65	Stem loosening	68	C2 Lima (Ti6Al4V, 12/14)	8.2
	M	Overweight	Primary instability	54	Cup loosening	54	SAM-FIT Lima (Ti6Al4V, 12/14)	0.7
Forte	F	Overweight	Dislocations	70	Stem loosening	71	CFP Link (Ti, 12/14)	1.9
	F	Unavailable	Pain	62	Stem loosening	68	CBC Mathys (Ti6Al4V, 12/14)	6.3
	M	Overweight	Unavailable	64	Stem loosening	64	QUADRA-S Medacta (Ti6Al7Nb, 12/14)	0.5
**Adapter**								
Cementless								
28 mm								
Delta	F	Overweight	Unavailable	66	Unavailable	67	CONUS Sulzer (Ti6Al7Nb, 12/14)	16.0
SS	F	Normal	Cup loosening	67	Stem loosening	67	STEM Cremascoli (Ti6Al4V, 12/14)	13.8
36 mm								
Delta	F	Overweight	Cup loosening	62	Stem loosening	68	CFP Link (Ti, 12/14)	11.9
	M	Overweight	Liner failure	67	Neck failure	73	RECTA Adler Ortho (Ti6Al4V, 12/14)	9.6
	M	Unavailable	Liner failure	46	Neck failure	50	APTA Adler Ortho (Ti6Al4V, 12/14)	8.5

[a] Loosening is aseptic unless otherwise specified. Pain indicates pain without loosening.
SS = stainless steel
